# REPURPOSING SODIUM-GLUCOSE CO-TRANSPORTER-2 INHIBITORS FOR GEROTHERAPY

**DOI:** 10.1093/geroni/igae098.0451

**Published:** 2024-12-31

**Authors:** Carolina Solis-Herrera, Venugopal Venna, Juneyoung Lee

**Affiliations:** University of Texas, San Antonio, Texas, United States; UT Health Houston, Texas, United States; UT Health Houston, Texas, United States

## Abstract

Sodium-glucose co-transporter-2 inhibitors (SGLT2i) are drugs used to treat T2DM, offering benefits such as reduced HbA1c, weight loss, and improved insulin sensitivity/β-cell function. Additionally, they provide significant cardio-renal benefits, although the exact mechanisms are not fully understood. The ketone hypothesis suggests that SGLT2i may shift metabolism towards utilizing ketone bodies instead of fatty acids, contributing to their therapeutic effects. Previous research has shown that SGLT2i can increase plasma ketones 2-4-fold. One intriguing area of research is the potential use of SGLT2i as agents for late-aging and longevity. T2DM exacerbates aging-related biomarkers such as senescence, chronic inflammation, oxidative stress, DNA damage, and mitochondrial dysfunction. Preclinical studies have demonstrated that SGLT2i possess anti-inflammatory, anti-oxidative, and anti-senolytic properties, suggesting they may improve health span and lifespan, particularly in patients with chronic inflammatory diseases like T2DM and obesity. However, clinical research in this area is still limited. Preliminary data from our group suggests that in older, obese subjects with prediabetes, treatment with an SGLT2 inhibitor for 12 weeks led to a significant decrease in global and adipose senescent and inflammatory profiles. This effect, correlated with increased plasma ketones, indicates a potential mechanism by which SGLT2i may modulate aging processes and improve symptoms of age-related diseases. Here, we will discuss this and other current studies that support the notion that SGLT2i may contribute to the increase of both healthspan and lifespan.

